# Role of sacubitril-valsartan in the prevention of atrial fibrillation occurrence in patients with heart failure: A systematic review and meta-analysis of randomized controlled trials

**DOI:** 10.1371/journal.pone.0263131

**Published:** 2022-01-26

**Authors:** Xuehui Liu, Hongjun Liu, Lijun Wang, Lei Zhang, Qiang Xu

**Affiliations:** 1 Department of Cardiology, Yichang Hospital of Traditional Chinese Medicine, Yichang, China; 2 Traditional Chinese Medicine Hospital of China Three Gorges University, China Three Gorges University, Yichang, China; 3 Clinical Medical College of Traditional Chinese Medicine, China Three Gorges University, Yichang, China; Karolinska Institutet, SWEDEN

## Abstract

**Objective:**

Heart failure (HF) and atrial fibrillation (AF) are often coexisting. They have common risk factors and pathophysiologic mechanisms. Sacubitril/valsartan has shown efficacy and tolerability in patients with HF. Thus, the study was performed to evaluate the impact of sacubitril/valsartan on AF occurrence in patients with HF.

**Methods:**

The Embase and PubMed were searched from their inception date to June 2021 for all relevant randomized controlled trials (RCTs) evaluating the efficacy of sacubitril/valsartan in HF. The outcome of interest was the AF occurrence during the follow-up. Relative risks (RRs) with 95% confidence intervals (CIs) were pooled using a random-effects model.

**Results:**

Six trials involving a total of 15,512 patients were included (7,750 randomized to sacubitril/valsartan and 7,762 to control). All trials were randomized, double-blind, and active-control. There was no significant difference in the prevention of AF occurrence between the sacubitril/valsartan group and the control group (enalapril or valsartan) in patients with HF (RR 1.07, 95%CI 0.95 to 1.19; I^2^ 4%).

**Conclusions:**

Sacubitril/valsartan was similar to either enalapril or valsartan in preventing the occurrence of AF in patients with HF.

## 1. Introduction

Atrial fibrillation (AF) is the most common sustained cardiac arrhythmia in patients with heart failure (HF) [1]. HF and AF are often coexisting, because they share common risk factors and pathophysiologic mechanisms [2]. The structural and neurohormonal changes in HF increase the risk for the development of AF, both in HF with reduced ejection fraction (HFrEF) and preserved ejection fraction (HFpEF) [2, 3]. AF is both a consequence and cause of HF, with complex interactions leading to impairment of cardiac function [2]. Regardless of which comes first, patients with concomitant HF and AF have a significant worse prognosis [4].

Drugs that are used to treat HF and might also impact AF. Angiotensin converting enzyme inhibitors (ACEIs) and angiotensin receptor blockers (ARBs) are the cornerstone of HFrEF therapy [5]. Previous meta-analysis demonstrated that ACEIs/ARBs can reduce the risk of incident AF in patients with HF [6]. Sacubitril/valsartan is an angiotensin receptor neprilysin inhibitor (ARNI). In the PARADIGM-HF trial, sacubitril/valsartan was superior to enalapril in reducing the risk of cardiovascular death and of hospitalization for HF [7]. Given its advantages, ARNI therapy is recommended as a replacement for patients with HFrEF who have been previously exposed to ACEIs or ARBs [5, 8, 9]. However, it is not known whether ARNI therapy will reduce AF occurrence in patients with HF. Thus, we performed this systematic review and meta-analysis to assess the effect of sacubitril/valsartan on the occurrence of AF in patients with HF.

## 2. Methods

### 2.1 Search strategy

We conducted a systematic search using Embase and PubMed from their inception to June 2021. No language restriction was applied. The search terms used were “sacubitril”, “sacubitril-valsartan”, “LCZ696”, “neprilysin”, “heart failure”, “randomized controlled trial”, “randomised controlled trial”, “randomly” and “trial”. We also manually screened the reference lists of all included trials and relevant reviews to identify other potentially eligible articles. This meta-analysis was conducted according to the Preferred Reporting items for Systematic Reviews and Meta-analysis (PRISMA) statement [10].

### 2.2 Study selection

Studies were included if they met all of the following criteria: 1) randomized controlled trial; 2) comparing sacubitril/valsartan with ACEI/ARB/placebo; 3) enrolling patients with HF; 4) reporting data on AF occurrence during follow-up. The exclusion criteria were as follow: 1) articles related to the same trial; 2) observational studies, case reports, abstracts, and reviews.

### 2.3 Data extraction and quality assessment

Data extraction was performed independently by two reviewers. We extracted the following information from each included study: first author, publication year, demographic characteristics, number of patients, number of events, and follow-up duration. When duplicate reports of the same trial were found, data from the most complete dataset were extracted for analysis. Disagreements were resolved by consensus.

The outcome of interest was the occurrence of AF during follow-up. However, AF was not a pre-specified endpoint in all of the included trials. The number of AF occurrence in each trial was extracted according to adverse event data in ClinicalTrials.gov. In addition, the occurrence of atrial flutter in each trial was also extracted.

The methodological quality of included trials was assessed by using the Cochrane Collaboration Risk of Bias Tool (Reviews Manager 5.4).

### 2.4 Statistical analysis

For dichotomous data, we calculated relative risks (RRs) with 95% confidence intervals (CIs). We pooled outcome data using random-effects models regardless of heterogeneity. Heterogeneity across studies was assessed by using the I^2^ statistic [11]. An I^2^ value greater than 50% indicates significant heterogeneity. Subgroup analyses were conducted to examine the influence of various factors on the overall pooled estimate. Publication bias was assessed by using funnel plot. A two-sided *P*-value < 0.05 was considered statistical significance. All analyses were performed using Review Manager software (RevMan version 5.4; The Nordic Cochrane Centre, Cochrane Collaboration).

## 3. Results

### 3.1 Study search

**Fig 1:**
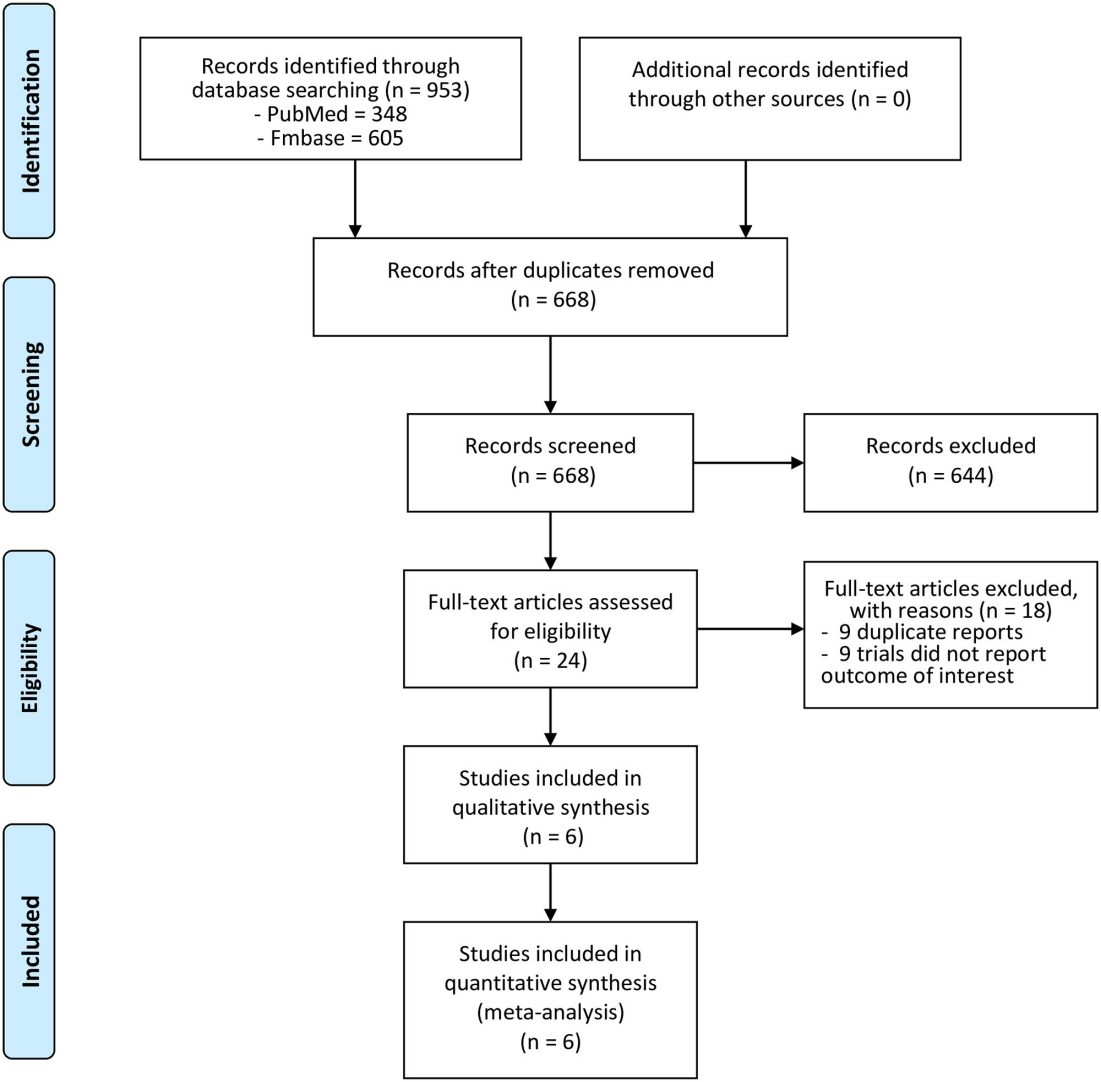
Flow diagram of search strategy.

Our initial search yielded a total of 953 studies, of which 285 were duplicates. After screening the titles and abstracts, 24 studies were considered potentially eligible. After reviewing the full text and the adverse event data in ClinicalTrails.gov, six trials [7, 12–16] were included in our meta-analysis. The study selection process is showed in Fig 1.

### 3.2 Study characteristics and quality assessment

The baseline characteristics of the six included RCTs are summarized in Table 1. All trials were randomized, double-blind, and active-control. The trials were published between 2012 and 2019. A total of 7,756 patients received sacubitril/valsartan, 5,208 patients received enalapril, and 2,554 patients received valsartan. Four trials [7, 13, 15, 16] used enalapril as a comparator, the other trials [12, 14] used valsartan as a comparator. Three trials [7, 13, 16] randomized a total of 9,525 patients with HFrEF, two trials [12, 14] randomized 5,123 patients with HFpEF, and one [15] randomized 881 patients with acute decompensated HF. The sample size ranged from 301 to 8,442. The follow-up duration ranged from 8 weeks to 35 months. All included trials did not describe the definition of AF and the methods used to document AF.

**Table 1:** Characteristics of included studies.

Study, year	Sample size	Type of HF	NYHA class	Mean age (years)	Female (%)	Mean LVEF	Follow-up	Comparator	History of AF		Occurrence of AF	
									S/V	Control	S/V	Control
PARAMOUNT, 2012 [12]	301	HFpEF	I-III	71.1	170 (56.5)	58%	36 weeks	Valsartan	40	45	3	9
PARADIGM-HF, 2014 [7]	8442	HFrEF	I-IV	63.8	1,832 (21.7)	29.5%	27 months	Enalapril	1517	1574	267	250
EVALUATE-HF, 2019 [13]	464	HFrEF	I-III	67.3	109 (23.5)	33.5%	12 weeks	Enalapril	NA	NA	2	0
PARAGON-HF, 2019 [14]	4822	HFpEF	I-IV	72.8	2479 (51.4)	57.6%	35 months	Valsartan	775	777	410	384
PIONEER-HF, 2019 [15]	881	ADHF	I-IV	62	246 (55.8)	25%	8 weeks	Enalapril	NA	NA	4	3
OUTSTEP-HF, 2020 [16]	619	HFrEF	II-IV	66.9	132 (21.3)	NA	12 weeks	Enalapril	147	122	8	4

AF, atrial fibrillation; HF, heart failure; HFrEF, heart failure with reduced ejection fraction; HFpEF, heart failure with preserved ejection fraction; LVEF, left ventricular ejection fraction; NA, data not available; NYHA, New York Heart Association; S/V, sacubitril/valsarta

**Fig 2:**
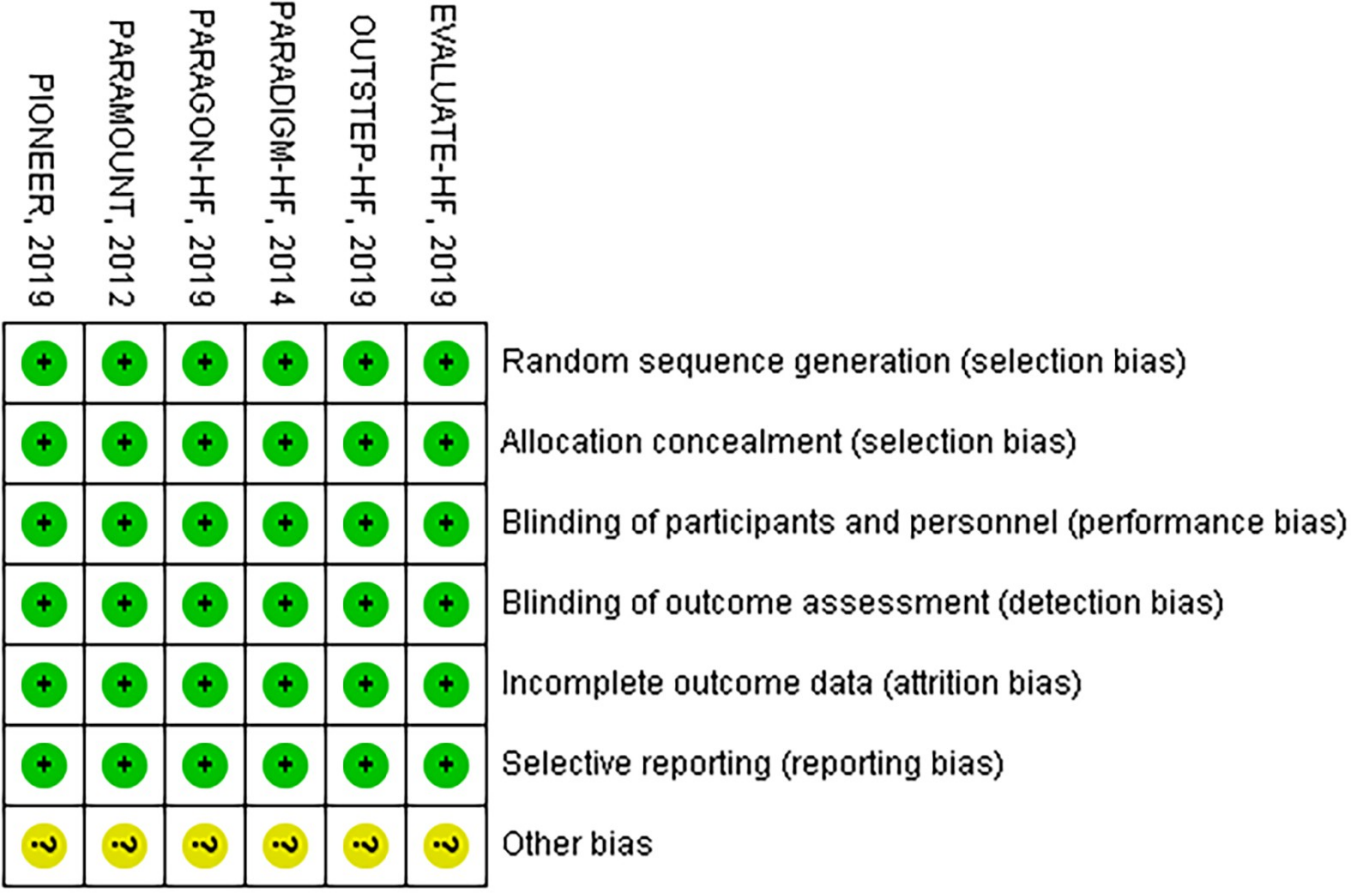
Methodological quality of included studies.

All included trials had a low risk of random sequence generation, allocation concealment, incomplete outcome data, selective reporting bias, except for unclear other bias (Fig 2).

### 3.3 Effect of sacubitril/valsartan on the occurrence of AF

**Fig 3:**
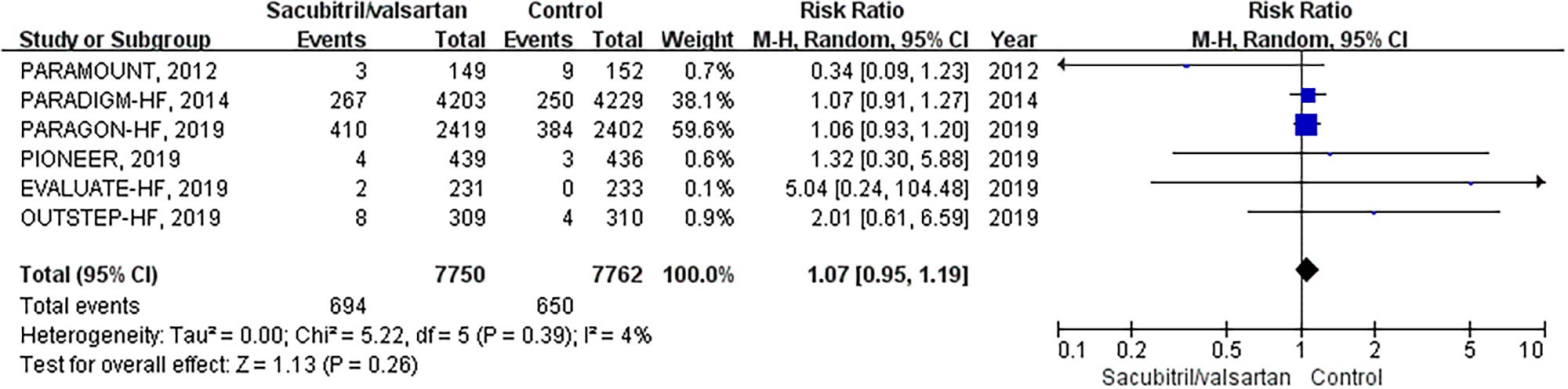
Forest of AF occurrence comparison: Sacubitril/valsartan group vs. control group.

AF occurred in 694 of 7750 (9.0%) patients who were on sacubitril/valsartan and 650 of 7762 (8.4%) patients among those in the control group. The risk of AF occurrence between the sacubitril/valsartan group and the control group did not show statistical significance (RR 1.07; 95% CI: 0.95–1.19; P = 0.26; Fig 3). There was no heterogeneity across the studies (I^2^ = 4%, P = 0.39). Publication bias analysis was not conducted because the number of trials was too small (<10) to detect an asymmetric funnel.

Three trials [7, 14, 15] reported the number of atrial flutter during follow-up. Atrial flutter occurred in 44 of 7,061 (0.6%) patients who were on sacubitril/valsartan and 42 of 7,067 (0.6%) patients among those in the control group. There was no significant difference in the pooled occurrence of atrial flutter (RR 1.01; 95% CI: 0.55–1.86; P = 0.97; I^2^ = 34%). In addition, no significant difference was observed in preventing AF and atrial flutter between the sacubitril/valsartan and the control group (RR 1.07; 95% CI: 0.97–1.18; P = 0.84; I^2^ = 0%).

Subgroup analyses were performed according to HF phenotype (HFrEF vs. HFpEF), comparator (enalapril vs. valsartan), and follow-up duration (<1 year vs. >1 year). The HF phenotype, comparator, and duration of follow-up did not appear to influence the effect of therapy (Table 2).

**Table 2:** The pooled effects of sacubitril/valsartan in different patient populations.

	Categorical data	RR (95%CI)	Heterogeneity test (I^2^, P)
Type of chronic HF	HFrEF [7, 13, 16]	1.10 (0.90, 1.34)	1%, P = 0.36
	HFpEF [12, 14]	0.72 (0.25, 2.08)	66%, P = 0.08
Control group	Enalapril [7, 13, 15, 16]	1.10 (0.93, 1.29)	0%, P = 0.56
	Valsartan [12, 14]	0.72 (0.25, 2.08)	66%, P = 0.08
Follow-up duration	> 1 years [7, 14]	1.07 (0.96, 1.18)	0%, P = 0.90
	< 1 years [12, 13, 15, 16]	1.14 (0.41, 3.14)	42%, P = 0.16

HF, heart failure; HFrEF, heart failure with reduced ejection fraction; HFpEF, heart failure with preserved ejection fraction; CI: confidence interval; RR, relative risks

## 4. Discussion

In this meta-analysis of six RCTs, we evaluated the effect of sacubitril/valsartan in the prevention of AF occurrence in patients with HF. The pooled effects demonstrate that sacubitril/valsartan is similar to either enalapril or valsartan in the prevention of AF occurrence in patients with HF.

It is well known that HF patients are at increased risk of AF [17]. Activation of renin-angiotensin-aldosterone system (RAAS) and neurohormonal pathways have been widely explored in HF, and the majority of drug therapies target these mechanisms [2]. Previous studies showed that ACEIs and ARBs could reduce the risk of AF occurrence when compared with placebo, with RR of 0.79 (95% CI: 0.62–1.00) and 0.78 (95%CI: 0.66–0.92), respectively [6, 18]. A post-hoc analysis of the Val-HeFT (Valsartan Heart Failure Trial) showed that adding valsartan to prescribed therapy for HF reduces the incidence of AF by 37% [19]. Data from the CHARM (Candesartan in Heart Failure-Assessment of Reduction in Mortality and morbidity program) demonstrated that candesartan could reduce the risk of new-onset AF in patients with HF [20]. A retrospective study of SOLVD including 391 patients with mean left ventricular ejection fraction < 30%, those receiving enalapril treatment markedly reduce the risk of development of AF during a mean follow-up of 2.9 years [1]. In the PARADIGM-HF trial [7], new-onset AF developed in 84 patients (3.1%) in the LCZ696 group and 83 patients (3.1%) in the enalapril group (P = 0.84). Sacubitril/valsartan has proven to be superior to ACEI/ARB in reducing cardiovascular death and hospitalization for HFrEF [8]. Given its advantages, current HF guidelines have incorporated sacubitril/valsartan into their recommendations for HFrEF [5, 8, 9]. Cross-trial comparisons are inherently limited because of differences in the trial designs and trial participants; however, the magnitude of benefit, relative to sacubitril/valsartan, appears to be at least as well as ACEIs/ARBs, all of which have been associated with a reduction in the risk of AF occurrence.

There are several mechanisms that sacubitril/valsartan may impact the development or occurrence of AF. Left atrial enlargement and cardiac hypertrophy are strongly associated with the initiation and maintenance of AF. In PARAMOUNT trial, sacubitril/valsartan significantly reduced left atrial volume [12]. A meta-analysis showed that sacubitril/valsartan significantly improved hypertrophy compared with ACEIs/ARBs in HFrEF [21]. Fibrosis is an important pathophysiological basis of cardiac remodelling [17]. The activation of RAAS results in the stimulation of fibrosis [22]. Therefore, RAAS inhibitors are involved in the upstream treatment of AF. Von Lueder et al. reported that sacubitril/valsartan attenuated cardiac remodelling and dysfunction after MI due to the inhibition of cardiac fibrosis and cardiac hypertrophy [23]. Suematsu et al. showed that LCZ696 was superior to valsartan with regard to the inhibition of cardiac fibrosis against HFrEF with a diabetes model [24]. Neprilysin degrades biologically active natriuretic peptides, which have been proven to inhibit fibrotic responses to prevent structural and electrical remodelling [25]. In animal models, it have been reported that sacubitril/valsartan reduce AF susceptibility by inhibiting atrial fibrosis [22, 25, 26]. All the above studies suggest that the anti-arrhythmic actions of sacubitril/valsartan might be mediated by reversing cardiac remodelling.

Because of the worse clinical outcomes of HF patients who develop AF, an effective management of upstream may prevent or delay the development of AF [17]. According to our study, it is premature to recommend sacubitril/valsartan solely for the prevention of AF, but our findings raise the possibility of an added benefit in HF patients receiving ARNI therapy.

There are several limitations in our meta-analysis that should be noted. First, AF was not a pre-specified endpoint in all included trials. The definition of AF and the methods used to document AF were not reported. The AF occurrence was calculated by reported adverse events. It is difficult to exclude the fact that some of the patients had asymptomatic AF that converted spontaneously. Secondly, there are some differences among the study designs, patient populations, and control drugs in the six trials that may affect the pooled effects. Thirdly, unpublished data were not included. Finally, all included trials were sponsored by Novartis. This might introduce high risk of bias as drug studies sponsored by manufacturing companies have more favorable efficacy results and conclusions than studies sponsored by other sources [27]. These limitations should be considered when interpreting the results.

## 5. Conclusions

Sacubitril/valsartan was similar to either ACEI or ARB in preventing the occurrence of AF for HF. Yet the PARADIGM-HF and PARAGON trials were not designed to show an impact on the occurrence of AF and further trials are required to assess the efficacy whether sacubitril/valsartan might impact the occurrence or development of AF.

## Supporting information

S1 Checklist PRISMA 2009 checklist. (DOC)

## Abbreviations

ACEI: angiotensin converting enzyme inhibitor
AF: atrial fibrillation
ARB: angiotensin receptor blocker
ARNI: angiotensin receptor neprilysin inhibitor
CI: confidence interval
HF: heart failure
HFpEF: heart failure with preserved ejection fraction
HFrEF: heart failure with reduced ejection fraction
RCT: randomized controlled trial
RR: relative risk
